
JOURNAL INFORMATION
==============================
NLM Title Abbreviation: ChemistryOpen
Journal ID: ChemistryOpen
NLM Journal ID: 101594811
Journal ID: open

ChemistryOpen

EISSN: 2191-1363
Publisher: Wiley

ARTICLE INFORMATION
==============================
PMCID: PMC3594584
DOI: 10.1002/open.201390004
Article version: 1
Subjects: Masthead

Masthead: ChemistryOpen 1/2013

Print publication date: 2013 Feb
Electronic publication date: 2013 Feb 27
Volume: 2
Issue: 1
First page: 9
Last page: 9
Copyright: Copyright © 2013 WILEY-VCH Verlag GmbH & Co. KGaA, Weinheim
Copyright year: 2013
License: Re-use of this article is permitted in accordance with the Creative Commons Deed, Attribution 2.5, which does not permit commercial exploitation.
License URL: https://creativecommons.org/licenses/by/2.5/

==============================
### Article Publication Charges

All articles published by *ChemistryOpen* are fully open access: immediately freely available to read, download and share. To cover the cost of publishing, *ChemistryOpen* charges a publication fee.
*ChemistryOpen's* publication charge for authors submitting a **Full Paper** or **Communication** to the journal is € 2500. Authors wishing to publish a **Thesis Summary** in the *ChemistryOpen* Thesis Treasury will be charged € 500.
Authors who provide proofs of membership (membership number) to a ChemPubSoc Europe society receive a 20% discount on the associated publication charge.
Further discounts might be available through your funding agency or institution. Please visit the journal homepage for further details of the Article Publication Charges.

### Professional Reprint Orders

To order professional reprints, e-prints, posters and more, please contact Carmen Leitner:
Fax: (+49) 6201-606-226
E-mail: chem-reprints@wiley-vch.de

Registered names, trademarks, etc., used in this journal, even when not marked as such, are not to be considered unprotected by law.

### For the USA and Canada

*ChemistryOpen* (ISSN 2191-1363) is published bimonthly by Wiley-VCH, PO Box 191161, 69451 Weinheim, Germany.
Wiley's Corporate Citizenship initiative seeks to addresss the environmental, social, economic, and ethical challenges faced in our business and which are important to our diverse stakeholder groups. We have made a long-term commitment to standardize and improve our efforts around the world to reduce our carbon footprint. Follow our progress at www.wiley.com/go/citizenship

### Open Access License and Copyright

All *ChemistryOpen* articles are published under the terms of the Creative Commons Attribution Non Commercial License which permits use, distribution and reproduction in any medium, provided the original work is properly cited and is not used for commercial purposes.
Copyright on any research article published by a Wiley-VCH Open Access journal is retained by the author(s).
Authors grant Wiley-VCH a license to publish the article and identify itself as the original publisher.
Authors also grant any third party the right to use the article freely as long as its integrity is maintained and its original authors, citation details and publisher are identified.
The Wiley-VCH Open Access Agreement formalizes these and other terms and conditions of publishing research articles.

Full details can be found at www.wileyopenaccess.com

**How to cite**: To make sure that references to this journal are correctly recorded and resolved (for example in CrossRef or ISI Web of Science), please use the following abbreviated title in any citations: "*ChemistryOpen*" (one word, no spaces or punctuation)

Access *ChemistryOpen* from your desktop.

### Homepage:

www.chemistryopen.org

Articles are available online in EarlyView well before the issue is published, through the Wiley Online Library service.

WILEY ONLINE LIBRARY
wileyonlinelibrary.com

### Publication:

6 online issues in 2013
ISSN: 2191-1363

A Journal of
ChemPubSoc Europe

**Typesetting:**
Triltsch print und digitale medien
Johannes-Gutenberg-Strasse 1–3
97199 Ochsenfurt-Hohestadt
www.triltsch.de

Printed in the
Federal Republic of Germany

Authors who wish to publish in *ChemistryOpen* should consult the current "Notice to Authors", which appears on the *ChemistryOpen* homepage at http://www.chemistryopen.org, before they draft their manuscript.

© 2012 Wiley-VCH Verlag GmbH & Co. KGaA, Weinheim
